# Determining hemodilution in diagnostic bone marrow aspirated samples in plasma cell disorders by next-generation flow cytometry: Proposal for a bone marrow quality index

**DOI:** 10.1038/s41408-023-00951-2

**Published:** 2023-12-01

**Authors:** Jón Þórir Óskarsson, Sæmundur Rögnvaldsson, Sigrun Thorsteinsdottir, Thor Aspelund, Steinar Bragi Gunnarsson, Guðlaug Katrín Hákonardóttir, Guðrún Ásta Sigurðardóttir, Ásdís Rósa Þórðardóttir, Gauti Kjartan Gíslason, Andri Ólafsson, Jón Kristinn Sigurðsson, Elías Eyþórsson, Ásbjörn Jónsson, Brynjar Viðarsson, Páll Torfi Önundarson, Bjarni A. Agnarsson, Róbert Pálmason, Margrét Sigurðardóttir, Ingunn Þorsteinsdóttir, Ísleifur Ólafsson, Stephen Harding, Juan Flores-Montero, Alberto Orfao, Brian G. M. Durie, Thorvardur Jon Love, Sigurdur Yngvi Kristinsson

**Affiliations:** 1 Faculty of Medicine, University of Iceland, Reykjavík, Iceland; 2 Landspítali University Hospital, Reykjavík, Iceland; 3 Department of Hematology, Rigshospitalet, Copenhagen, Denmark; 4 Public Health Sciences, University of Iceland, Reykjavík, Iceland; 5 Akureyri Hospital, Akureyri, Iceland; 6 Department of Hematology, Oncology and Radiation Physics, Skåne University Hospital, Lund, Sweden; 7 The Binding Site Inc., Birmingham, West Midlands UK; 8 Cancer Research Center (IBMCC, USAL-CSIC), Department of Medicine and Cytometry Service (NUCLEUS), University of Salamanca; Biomedical Research Institute of Salamanca (IBSAL) and CIBERONC, Salamanca, Spain; 9 Cedars-Sinai Samuel Oschin Cancer Center, Los Angeles, CA USA

**Keywords:** Myeloma, Myeloma, Cancer microenvironment

## Abstract

Hemodilution of bone marrow (BM) aspirates is a limitation of multiparameter flow cytometry (MFC) in plasma cell disorders. There is a need for a validated approach for assessing sample quality and the distribution of non-plasma cell BM populations by MFC could provide a solution. We evaluated BM-associated cell populations, assessed by next-generation flow cytometry (NGF) and white blood cell (WBC) count in 351 BM aspirated samples from 219 participants with plasma cell disorders in the Iceland Screens, Treats, or Prevents MM study (iStopMM), as markers of hemodilution by their discriminatory ability between first and (generally more hemodiluted) second pull BM aspirated samples. The most discriminating markers were used to derive a novel BM quality index (BMQI). Nucleated red blood cells and myeloid precursors provided the greatest discriminatory ability between first vs second pull samples (area under the curve (AUC): 0.87 and 0.85, respectively), significantly better than B cell precursors (AUC = 0.64; *p* < 0.001), mast cells (AUC = 0.65; *p* < 0.001), and the BM WBC count (AUC = 0.77; *p* < 0.05). We generated a novel BMQI that is intrinsic to current NGF protocols, for evaluating quality of diagnostic BM samples and suggest the use of a BMQI scoring system for interpreting results and guiding appropriate actions.

## Introduction

Multiple myeloma (MM) is a hematological malignancy, characterized by the expansion and accumulation of malignant plasma cells in the bone marrow (BM) [1]. MM is always preceded by asymptomatic precursor conditions, monoclonal gammopathy of undetermined significance (MGUS) and the more advanced precursor state smoldering MM (SMM), with an annual risk of progression to symptomatic MM of approximately 1% and 10%, respectively [2–4]. Historically, MM has been considered an incurable disease with a poor prognosis. However, recent advances in treatment options for MM, including novel therapeutic agents, have dramatically changed the clinical course and outcome of MM, leading to an improved survival [5–7]. Consequently, deeper responses are observed than before and monitoring of measurable residual disease (MRD) by high-sensitive techniques has become common in patients who are in conventional complete response [8–10].

Together with next-generation sequencing (NGS) and positron emission tomography-computed tomography (PET-CT), next-generation flow cytometry (NGF) is currently recommended and used for MRD assessment of BM in MM. A major limitation of both NGS and NGF techniques for accurate quantification of BM plasma cells relies on the quality of the sample obtained and in particular the degree to which it is diluted with blood during the aspiration procedure [10, 11]. This is because plasma cells mostly reside in the BM and hemodilution of BM samples will therefore result in underestimation of plasma cell numbers that may lead to misdiagnosis in MRD assessment [12]. In fact, BM plasma cells have been shown to gradually decrease with sequential aspirate pulls, indicating that BM hemodilution is less pronounced in first pull samples (first few mL of aspirate) vs subsequently aspirated samples [13]. Thus, first pull BM samples or second pull BM samples obtained after repositioning the needle (whenever first pull samples are prioritized for other analysis), are currently recommended for MRD assessment in BM [14]. The sensitivity of the MRD assay in BM directly correlates with patient prognosis and outcome [9]. However, the reported sensitivity of the MRD techniques is dependent on the number of nucleated cells evaluated, regardless of whether they originate from the BM or not. Based on the lower frequency of tumor plasma cells in blood vs BM, hemodilution has a negative impact on the sensitivity of the MRD assays. An important advantage of MRD monitoring by NGF vs NGS relies on its ability to discriminate between tumor plasma cells and multiple other BM-associated cell populations in a high-sensitive and fully-standardized way, based on their unique immunophenotypes, as identified with the combination of markers used [10, 15–18]. NGF has therefore the potential to evaluate hemodilution based on the distribution of BM-derived vs blood-derived cell populations in the individual samples.

The potential of using BM-associated cell populations for intra-sample quality control in MRD analyses by NGF has been recognized [18]. Despite this, there is no established validated method for determining the degree of BM hemodilution for a given sample. This is due, at least in part, to the lack of BM hemodilution markers in general and the unavailability of pure BM controls. Mast cells have been initially proposed as a potential indicator of BM sample quality in NGF and used in early studies as an indicator of significant hemodilution [12, 14, 19]. In a recent paper, Puig et al. reported normal and treatment-specific reference values for mast cells, nucleated red cells and B-cell precursors for indication of hemodilution and warned about the potential false-negative results in MRD assessment [20]. However, in previous studies on assessment of BM hemodilution, the use of BM-associated cell populations has not included linkage to a reference of non-hemodiluted samples, relying on arbitrary inter-sample evaluation of hemodilution based on pre-selected BM-associated markers. To our knowledge, no study has reported a systematic comparison of the value of different BM-associated cell populations as markers of hemodilution in first vs second pull BM aspirates. There is an unmet need for an established approach for evaluating and reporting the quality of BM aspirated samples in MM and other monoclonal gammopathies.

Here, we evaluated the potential utility of non-plasma cell, BM-associated cell populations identified with the EuroFlow MRD panel for MM using NGF, to evaluate hemodilution based on comparison of their distribution in second vs first pull BM aspirates. Our goal was to build a predictive model for hemodilution that could be easily applied in the diagnosis and monitoring of MM patients by NGF.

## Methods

### Participants and samples

Participants were enrolled from the Iceland Screens, Treats, or Prevents MM study (iStopMM). The study is a population-based screening study for MM and its precursor diseases, followed by a randomized trial of different follow-up strategies. Briefly, a total of 80 579 Icelanders born before 1975 and who were alive in late 2016 (54.4% of the target population) gave their informed consent. Of them, 75 422 participants were screened for MM and other monoclonal gammopathies (MG) by serum protein electrophoresis (SPEP) and free light chain (FLC) assay. Those who tested positive were randomized to one of three study arms and two thirds called in to a clinical study center for further assessment and follow-up. The design and recruitment of the iStopMM study has been described in detail elsewhere [21]. All diagnosed cases of SMM and MM and a conveniency sample of participants with MGUS were eligible for a flow cytometry sub-study. As of May 2022, a total of 351 EDTA-anti-coagulated BM aspirated samples from 219 individuals had been collected as first pull (*n* = 204), second pull (*n* = 117), or paired first and second pull (*n* = 30 samples in total from 15 individuals) aspirated samples at a target volume of 2–4 mL (Table 1). BM sampling was performed by study nurses that had received specific BM sampling training, both locally and in an accredited facility in the United Kingdom (The Royal Marsden Hospital, London, UK). BM aspirated samples were passed through a 70 µm cell strainer and white blood cell (WBC) count measured using an ABX Micros ES 60 hematological analyzer (Horiba, Kyoto, Japan) before subsequent immunophenotypic analyses. To introduce controlled hemodilution for evaluating the performance of the BMQI, a fraction of the first pull BM samples (*n* = 12) was diluted with paired blood based on WBC count to obtain samples containing 70% and 30% cellularity of the original BM sample.

**Table 1:** Characteristics of BM aspirated samples.

Characteristic	All samples (*n* = 351)	MGUS (*n* = 128)	SMM (*n* = 186)	MM (*n* = 37)
age, median (range)	69 (43–89)	69 (43–88)	68 (44–88)	70 (49–89)
sex (%)	63% M - 37% F	43% M - 57% F	64% M - 36% F	78% M - 22% F
**Type, no. (%)**				
IgG	167 (47.6%)	68 (53.1%)	81 (43.5%)	18 (48.6%)
IgA	94 (26.8%)	30 (23.4%)	58 (31.2%)	6 (16.2%)
biclonal	30 (8.5%)	12 (9.4%)	15 (8.1%)	3 (8.1%)
light-chain^a^	60 (17.1%)	18 (14.1%)	32 (17.2%)	10 (27.0%)
**M-protein**^**b**^ **(g/dL), median (IQR)**	5.0 (1.0-11.0)	1.0 (0.2-3.0)	7.3 (4.0-13.0)	16.2 (8.0-53.0)
**FLC ratio**^**c**^**, median (IQR)**	15.6 (5.3-53.1)	3.2 (2.1-4.6)	19.1 (12.0-41.3)	183 (131-321)
**BMPC**^**d**^ **(%), median (IQR)**	13% (8%-26%)	8% (3%-8%)	18% (16%-26%)	51% (33%-66%)
**BM sample pull, no (%)**				
first	219 (62.4%)	44 (34.4%)	143 (76.9%)	32 (86.5%)
second	132 (37.6%)	84 (65.6%)	43 (23.1%)	5 (13.5%)

*MGUS* monoclonal gammopathy of undetermined significance, *SMM* smoldering multiple myeloma, *MM* multiple myeloma, *Ig* immunoglobulin, *BM* bone marrow, *M* monoclonal, *FLC* free light-chain, *BMPC* bone marrow plasma cells, *IQR* Interquartile range. ^a^Light-chain monoclonal gammopathy (abnormal light-chain levels and no detected M-protein); ^b^total serum M-protein concentration for individuals with identified M-protein; ^c^FLC ratio (involved/uninvolved) for individuals with light-chain monoclonal gammopathy; ^d^BMPC count by aspirate and biopsy with the higher value used (available for 336/351 samples).

### Immunophenotypic studies

The EuroFlow NGF MM-MRD panel was used for sample staining using antibody kits (Cytognos S.L., Salamanca, Spain) and the drop-in antibodies of CD138-BV421 (BD Biosciences, San Jose, CA, USA) and CD27-BV510 (BioLegend Inc., CA, USA), as described elsewhere [18]. Sample preparation and staining as well as instrument setup and calibration were performed according to the manufacturer’s specifications and the standardized EuroFlow protocols available at www.euroflow.org [22]. Samples were stained within 24 h of collection and measured in a single FACSCanto II flow cytometer (BD Biosciences). Flow cytometry data was analyzed using the Infinicyt software (Cytognos S.L.) based on the automated gating and identification (AG&I) tool and the EuroFlow MM-MRD reference database as recommended by EuroFlow. Results from automatic gating and classification were then manually reviewed. Events from tube 1 of the EuroFlow MM-MRD panel were then used to assess percentage values of myeloid precursors (CD117^+^, CD38^+^, CD45^lo^, and SSC^int/hi^), nucleated red cells (CD45^–^, CD38^–^, CD117^–^, and SSC^lo^), mast cells (CD117^hi^ and CD45^lo^), B cell precursors (CD19^+^, CD45^lo^, CD38^hi^, CD81^hi^, and CD27^–^), and plasma cells (CD38^hi^ and CD138^+^) (Supplementary Fig. 1). Because the bulk of tumor plasma cells varies widely in individuals with MM, SMM, and MGUS, non-plasma cell populations were assessed as percentages of total nucleated cells after excluding tumor plasma cells, whereas total plasma cells were assessed as percentages of total nucleated cells. The lower limit of quantification (LLOQ) was set at ≥50 cells per cell population and the LOQ was calculated from tube 1 by the following formula: 50/total number of nucleated cells.

### Study design

To investigate the potential relationship between the distribution of BM-associated cell populations and hemodilution, differences in the overall composition of first vs second BM aspirated sample pulls (reference for optimal and suboptimal samples, respectively) were used. As candidate markers for hemodilution we used those cell populations that are largely restricted to the BM and that are readily identified by the combination of markers used in assessing MRD in MM by NGF, including B cell precursors, myeloid precursors, nucleated red cells, and mast cells. In addition, the BM WBC count was also evaluated as an external reference of sample quality, independent of the NGF assay. The percentage of total plasma cells was used as indication of difference in the degree of hemodilution between groups and was evaluated for MGUS, SMM, and MM sample groups separately.

### Statistical methods

The Mann-Whitney *U* test or Wilcoxon signed-rank test were used to assess the statistical significance of differences between unpaired and paired first pull vs second pull BM samples, respectively. For the comparison of plasma cell numbers in our cohort, paired first vs second pull BM aspirated samples collected during the same BM aspiration procedure were used in order to limit donor variability due to the underlying disease/tumor load. In turn, for comparison of B cell precursor, myeloid precursor, nucleated red cell, and mast cell percentages as well as BM WBC count in first vs second pull samples, all BM samples (paired and unpaired) were used.

Receiver operating characteristic (ROC) curve analysis was used to assess the discriminatory power for the different BM-associated cell population percentages evaluated and the BM WBC count between first pull vs second pull samples. Area under the curve (AUC) was used to compare individual predictor variables as hemodilution markers and the DeLong’s test was used to assess statistical differences between ROC curves.

Donor variability is a limiting factor for predicting hemodilution based on cellular distribution. Therefore, we compared the differences in relative variability for predictor variables within the two groups of BM samples (first pull and second pull samples) using the respective coefficient of variation (CV), calculated by dividing the standard deviation (SD) by the mean value and multiply by 100.

For all statistical analysis and graphical presentation of data, the R statistical software (version 4.1.1; including packages pROC, MASS, and ggplot2) was used.

### Development and validation of the bone marrow quality index (BMQI)

We aimed to develop a multivariate model to predict the degree of hemodilution in BM aspirated samples. From our analysis of individual cell populations, myeloid precursors and nucleated red cells were chosen for the model due to their strong predictive performance for BM sample pull and comparatively lower relative variability. We employed linear discriminant analysis (LDA) to determine the linear combination of these populations for maximizing the between-group variance of first and second pull BM aspirated samples. Based on the coefficients derived from LDA analysis, the resulting BMQI incorporates the weighted contribution of each population, with higher BMQI values indicating less hemodilution. ROC analysis was used to assess the discriminatory ability of the BMQI, both in comparison to individual cell populations and separately within MGUS, SMM, and MM samples. The Kruskal-Wallis test was used to evaluate statistical significance of differences in BMQI between MGUS, SMM, and MM samples when stratified by pull. Pearson’s correlation analysis was utilized to examine the association between age and the BMQI.

The likelihood ratio (LR) for first vs second samples were used to derive corresponding BMQI values for classifying samples according to the observed probability of belonging to a class of first pull samples in a BMQI scoring system as followed: LR < 0.1: large decrease, LR 0.1–0.2: small to moderate decrease; LR 0.2-1: minimal decrease, LR 1–10: minimal to moderate increase, and LR > 10 large increase. The Kruskal-Wallis rank sum test and the Dunn multiple comparison test was then used to assess the statistical significance of differences observed in the percentage of BM plasma cells in samples grouped by ranges in BMQI, separately for MGUS and SMM groups. The Mann-Whitney *U* test was used to assess statistical significance of differences observed in the percentage of BM plasma cells in samples grouped according the derived BMQI value corresponding to a LR of 1, separately for MGUS, SMM, and MM groups.

The performance of the BMQI was assessed through three distinct sets of validation experiments using prospectively collected samples. Firstly, using a prospective series of paired first and second pull BM samples, collected during the same aspiration procedure. Secondly, in samples containing different proportions of BM sample-derived cells (100%, 70%, and 30%). These were experimentally diluted with peripheral blood (PB) based on cellularity (i.e., diluted samples contained pre-defined number of nucleated cells derived from the BM and blood samples). Lastly, in virtually diluted BM samples, simulated based on a pre-determined number of nucleated cells from flow cytometry data files of paired BM and PB samples (Supplementary methods). The pairwise Mann-Whitney *U* test and the Friedman test followed by the Nemenyi post hoc test were used to assess the statistical significance of differences observed between two and three groups in the validation series, respectively. Linear regression analysis was used to assess the relationship between the relative change in BMQI (independent variable) and the relative change in the percentage of total plasma cells (dependent variable) with dilution.

## Results

### Cohort description

A total of 351 BM aspirated samples (219 first pull and 132 second pull samples) collected from 219 individuals were analyzed by NGF. WBC count in BM was available for 336/351 (95.7%) of samples. The median (range) age at sampling was of 69 years (43-89 years) and 63.0% of samples were from males. Overall, 128 (36.5%) samples were from individuals diagnosed with non-IgM MGUS, 186 (53.0%) SMM, and 37 (10.5%) MM (diagnosis at the time of sampling). The median serum M-protein concentration in samples from individuals with identified M-protein was 1.0, 7.3, and 16.2 g/L and the median free light chain ratio (involved/uninvolved) among those with light-chain only involvement was 3.2, 19.1, and 183.3, for MGUS, SMM, and MM, respectively. The characteristics of the individuals included in the study cohort are further detailed in Table 1. The median (range) LOQ reached in NGF analysis was 1.4 × 10^−5^ (1.3 × 10^−4^–6.6 × 10^−6^). The number of plasma cells, myeloid precursors, and nucleated red cells were recorded within the quantifiable range of the NGF assay in all samples and B cell precursor counts in all but one sample, whereas mast cells were recorded above the LLOQ in 307/351 (87.5%) samples (89.5% of first pull and 84.1% of second pull samples; *p* = 0.14).

### Distribution of BM-associated cell populations in first and second pull BM samples

**Fig. 1:**
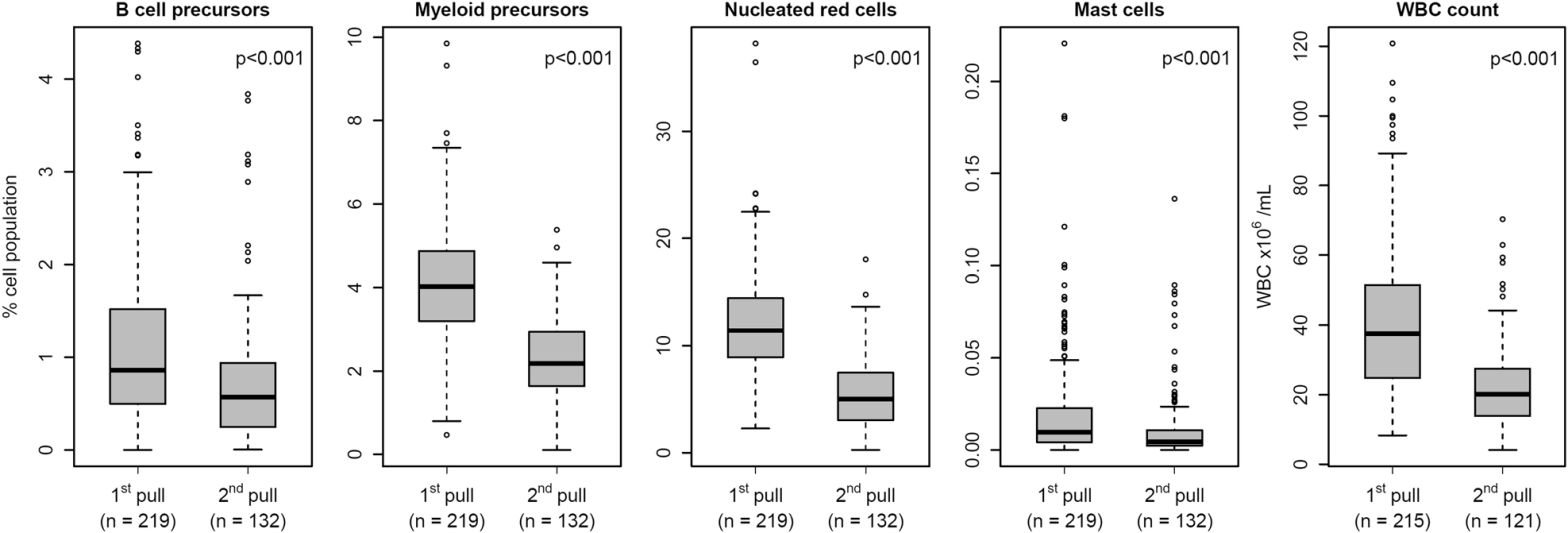
Distribution of bone marrow (BM)-associated cell populations and white blood cell (WBC) count in first and second pull BM aspirated samples. The figure shows the distribution of percentages (of normal cellularity) for B cell precursors, myeloid precursors, nucleated red cells, and mast cells, and BM sample WBC count in first pull vs second pull BM samples. Extreme outlier omitted from plot at 0.62% for mast cells for graphical presentation. *p* < 0.001 for all comparisons.

The percentage of plasma cells in first pull samples was significantly higher than in paired second pull samples taken at the same BM aspiration procedure (*n* = 15; *p* < 0.001) with a median fraction of plasma cell percentage between paired sample pulls (%plasma cells in second pull / %plasma cells in first pull) of 0.31 (range: 0.085-0.76). Similarly, a markedly higher median percentage of all BM-associated cell populations evaluated was observed in first pull samples (*n* = 219) compared to second pull samples (*n* = 132): B cell precursors, 0.86% vs. 0.56%; myeloid precursors, 4.02% vs 2.19%; nucleated red cells, 11.4% vs. 4.97%; and mast cells, 0.01% vs 0.004%, respectively (*p* < 0.001 for all) (Fig. 1 and Table 2). Similarly, a higher median WBC concentration was observed in first pull BM samples (*n* = 215) compared to second pull BM samples (*n* = 121): 37.5 vs. 20.1 WBC/mL, respectively (*p* < 0.001) (Fig. 1 and Table 2).

**Table 2:** Distribution of BM-associated cell population from the whole BM cellularity (apart from phenotypically abnormal plasma cells) and the WBC count in first and second pull BM aspirated samples.

Population	BM aspirate pull	Q25	Median (range)	Q75	CV, %	p-value
**B cell precursors, %**	first (*n* = 219)	0.50	0.86 (0.00–4.39)	1.52	80.8	7.9 × 10^–6^
	second (*n* = 132)	0.25	0.56 (0.004–3.84)	0.94	99.2	
**Myeloid precursors, %**	first (*n* = 219)	3.19	4.02 (0.47–9.85)	4.87	34.6	2.2 × 10^–16^
	second (*n* = 132)	1.65	2.19 (0.11–5.38)	2.93	46.0	
**Nucleated red cells, %**	first (*n* = 219)	8.92	11.4 (2.25–38.2)	14.4	40.1	2.2 × 10^–16^
	second (*n* = 132)	3.03	4.97 (0.25–18.0)	7.48	59.9	
**Mast cells, %**	first (*n* = 219)	0.004	0.01 (0.00–0.62)	0.02	215.3	2.2 × 10^–6^
	second (*n* = 132)	0.002	0.004 (0.00–0.14)	0.01	172.9	
**WBC count, 10**^**6**^**/mL**	first (*n* = 215)	24.8	37.5 (8.3–120.8)	51.5	53.8	2.2 × 10^–16^
	second (*n* = 121)	13.9	20.1 (4.1–70.3)	27.5	54.0	

*Q25 and Q75* Interquartile range, *CV* coefficient of variation.

### Ability of BM-associated cell populations to differentiate between BM sample pulls

**Fig. 2:**
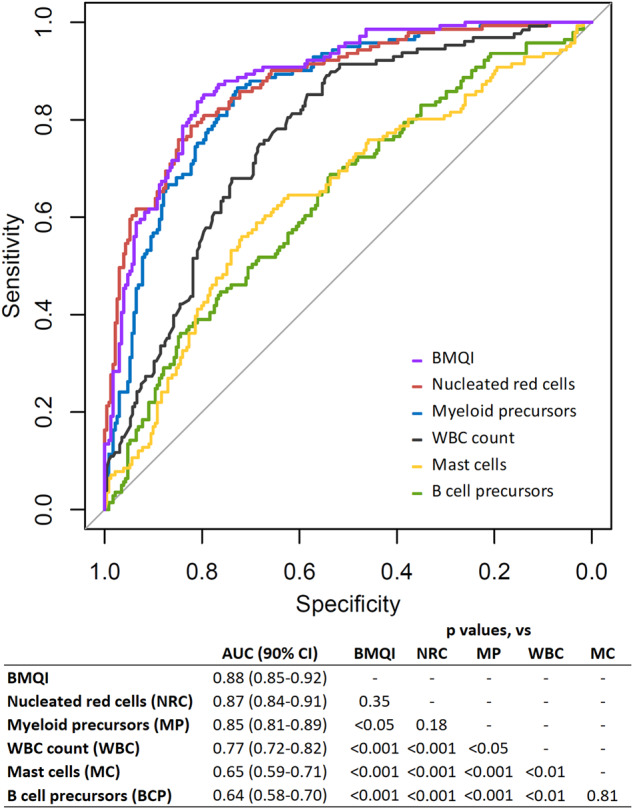
Discriminatory ability of the bone marrow (BM)-associated variables evaluated as hemodilution markers of BM sample pull. The figure depicts receiver-operating characteristic (ROC) curves for B cell precursors (in green), myeloid precursors (in blue), nucleated red cells (in red), mast cells (in yellow), BM sample white blood cell (WBC) count (in black), and bone marrow quality index (BMQI) (in violet) for discrimination between first and second pull BM aspirated samples. Sample sizes were *n* = 219 for first pull and *n* = 132 for second pull for cell populations and BMQI, and *n* = 215 for first pull and *n* = 121 for second pull for the WBC count. The table below the figures shows area under the curve (AUC) values with 90% confidence intervals (CI) for each ROC curve in the figure along with *p*-values indicating statistical significance of differences observed between ROC curves.

The percentage value of individual BM-associated cell populations showing the greatest discriminatory ability between first pull and second pull BM samples based on ROC analysis was observed for nucleated red cells (AUC = 0.87), followed by myeloid precursors (AUC = 0.85). Both populations were significantly more predictive of the BM sample pull compared to B cell precursors (AUC = 0.64; *p* < 0.001 vs both) and mast cells (AUC = 0.65; *p* < 0.001 vs both). When compared using the BM WBC count (AUC = 0.77) as reference, nucleated red cells and myeloid precursors were significantly more predictive of sample pull (*p* < 0.01 and *p* < 0.05, respectively), while B cell precursors and mast cells had a significantly lower predictive value of sample pull (*p* < 0.01 for both) (Fig. 2). The degree of relative variability among the percentage of the above cell populations and the WBC count in first pull BM samples, as assessed by the intra-group CV, was found to be lowest for myeloid precursors (34.6%), followed by nucleated red cells (40.1%), the WBC count (53.8%), B cell precursors (80.8%), and mast cells (215%). A similar trend was observed in second pull samples where increasing median intra-group CVs were found for myeloid precursors (46.0%), followed by the WBC count (54.0%), nucleated red cells (59.9%), B cell precursors (99.2%), and mast cells (172.9%) (Table 2).

### A novel BMQI for evaluating the degree of hemodilution in BM aspirated samples

Myeloid precursors and nucleated red cells had the greatest predictive value for sample hemodilution in second vs first pull BM and were included in the BMQI. The derived model for BMQI was (Eq. 1):

1 $${\rm{BMQI}}= \% {\rm{myeloid}}\,{\rm{precursors}}\times 0.37+ \% {\rm{nucleated}}\,{\rm{red}}\,{\rm{cells}}\times 0.14$$

Where the percentage values for myeloid precursors and nucleated red cells used in the equation are calculated from normal cellularity (total nucleated cells after excluding tumor plasma cells) and their respective coefficients represent the weighted contribution of each population to the BMQI.

The BMQI demonstrated a more robust predictive capability with a higher AUC value of 0.88 than both individual predictors. While its performance was significantly better than that of myeloid precursors (*p* < 0.05), the difference against nucleated red cells was not statistically significant (*p* = 0.35) (Fig. 2). The BMQI demonstrated consistent discriminatory ability for BM sample pull across MGUS, SMM, and MM samples with AUC values of 0.921, 0.882, and 0.875, respectively (*p* = 0.23 for MGUS vs SMM, *p* = 0.53 for MGUS vs MM, and *p* = 0.93 for SMM vs MM). Similarly, when stratified by BM sample pull, the BMQI distribution did not show significant variation between MGUS, SMM, and MM samples with median BMQI in first pull: 3.31, 3.14, and 2.87 (*p* = 0.20) and second pull: 1.58, 1.46, and 1.21 (*p* = 0.79), respectively. The correlation between age and BMQI was negligible and statistically insignificant (Pearsons’s r = -0.0044; *p* = 0.93; *n* = 351).

**Fig. 3:**
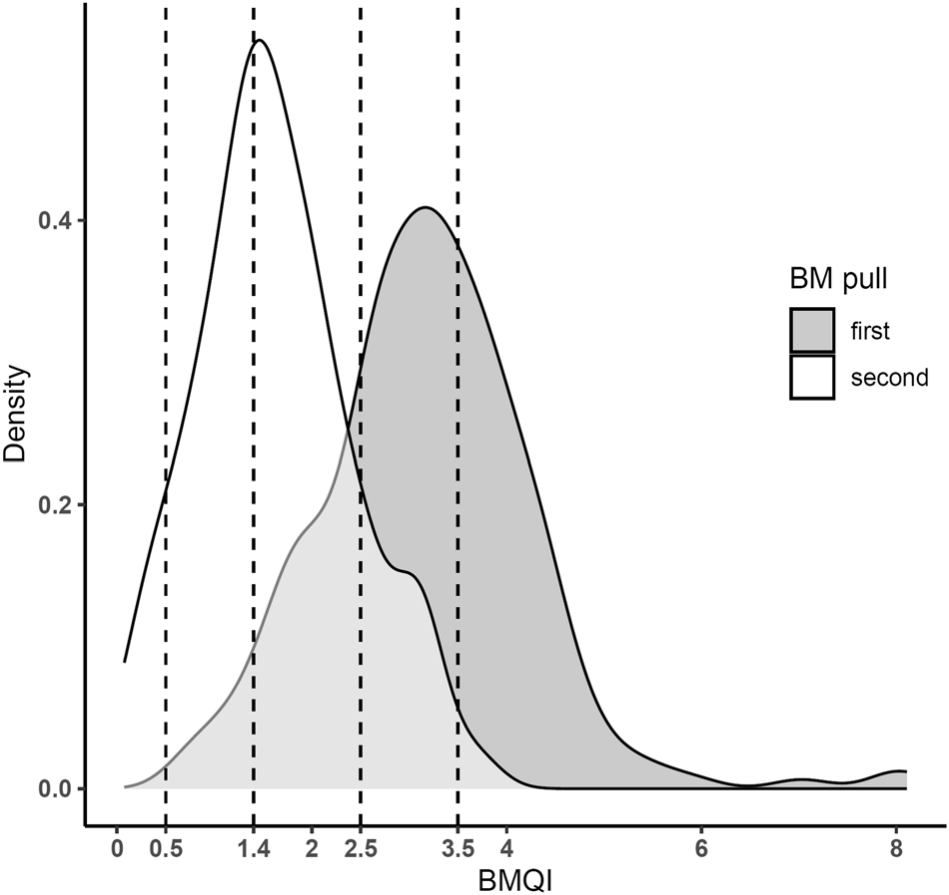
Distribution of the bone marrow quality index (BMQI) in first and second pull bone marrow (BM) aspirated samples. The figure shows the distribution of BMQI in first (in grey; *n* = 219) and second (in white; *n* = 132) pull BM aspirated samples as a density plot. The dotted lines represent the BMQI values for corresponding likelihood ratios of belonging to a class of first pull samples of 0.1 (BMQI = 0.5), 0.2 (BMQI = 1.4), 1 (BMQI = 2.5), and 10 (BMQI = 3.5) and used to classify samples in the BMQI scoring system.

**Fig. 4:**
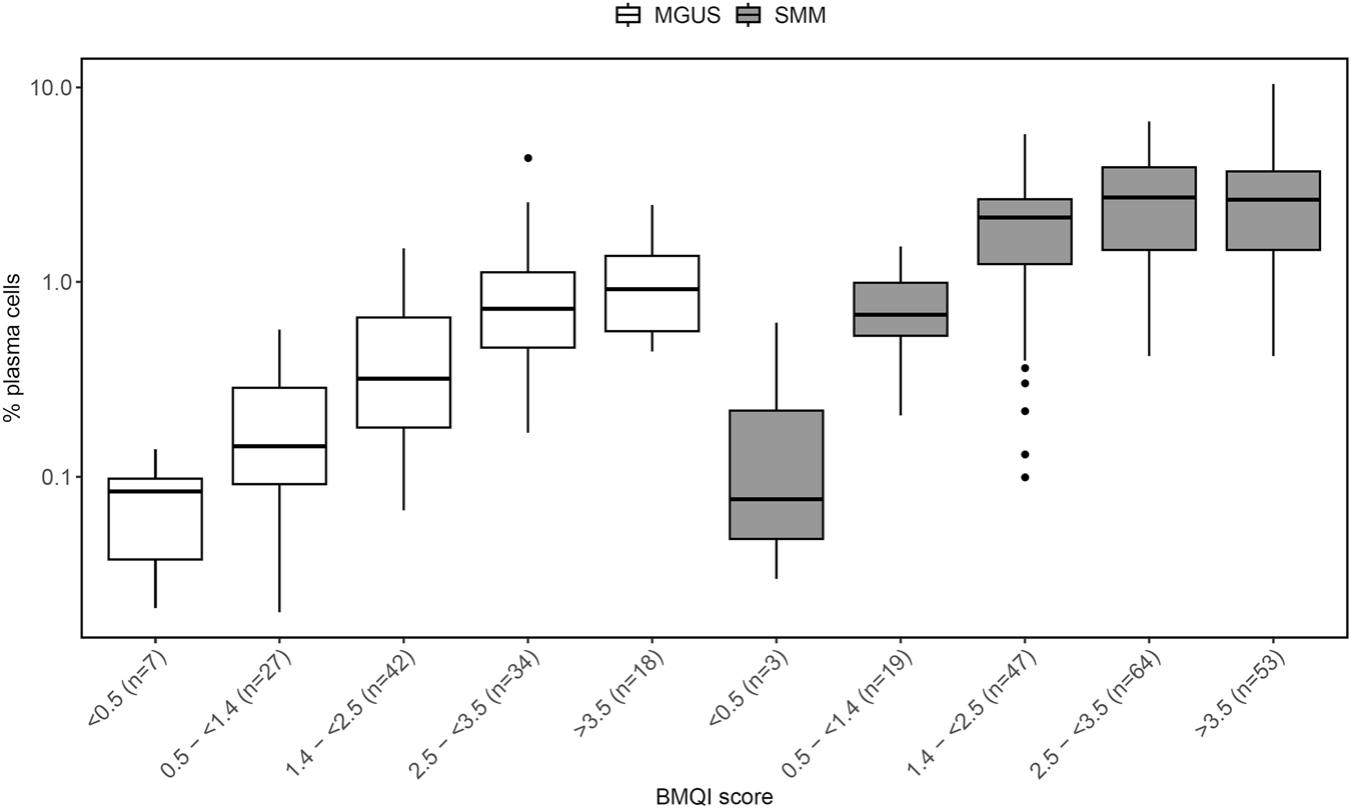
Distribution of the bone marrow (BM) plasma cell percentage in groups of monoclonal gammopathy of undetermined significance (MGUS) and smoldering multiple myeloma (SMM) samples defined by the bone marrow quality index (BMQI) score. The figure illustrates the total plasma cell percentage (of total nucleated cells) distribution in BM aspirated samples, grouped by BMQI score for MGUS on the left (in white) and SMM in the right (in gray). For MGUS, *p* < 0.05 (<0.5 vs. 1.4- < 2.5 and 1.4- < 2.5 vs. 2.5- < 3.5); *p* < 0.01 (1.4- < 2.5 vs. ≥3.5); and *p* < 0.001 (2.5-< 3.5 vs. ≥3.5 and 0.5- < 1.4 vs. 2.5- < 3.5 and ≥3.5). For SMM, *p* < 0.05 (<0.5 vs. 2.5- < 3.5); *p* < 0.01 (0.5- < 1.4 vs. 1.4- < 2.5); and *p* < 0.001 (0.5- < 1.4 vs. 2.5- < 3.5 and ≥3.5).

Next, we used BMQI values corresponding to the LR of 0.1, 0.2, 1, and 10 of belonging to a class of first pull samples to group samples according to predicted hemodilution ranging from severe hemodilution to negligible hemodilution as followed: BMQI < 0.5, 0.5- < 1.4, 1.4- < 2.5, 2.5- < 3.5 and ≥3.5 (Fig. 3). A gradual increase in the percentage of plasma cells was observed with increased BMQI score from <0.5 to ≥3.5 in MGUS (median: 0.084%, 0.14%, 0.32%, 0.73%, and 0.92%; *p* < 0.001) and SMM samples (median: 0.077%, 0.68%, 2.2%, 2.7%, and 2.7%; *p* < 0.001) (Table 3 and Fig. 4). Similarly, the BM sample WBC count was gradually increased in parallel to the BMQI score (*p* < 0.001) (Table 3). A cutoff in BMQI of 2.5 was found to discriminate between first and second pull samples with high accuracy (77.2% sensitivity and 84.1% specificity) and samples with a BMQI ≥ 2.5 had a significantly higher percentage of plasma cells compared to samples with a BMQI < 2.5 in the MGUS, SMM, and MM groups separately, with median plasma cell percentages of 0.77% vs. 0.21% (*p* < 0.001) in MGUS, of 2.7% vs. 1.4% (*p* < 0.001) in SMM, and of 7.9% vs. 3.4% (*p* < 0.05) in MM.

**Table 3:** Distribution of BM sample pull, WBC count, and plasma cell percentages in groups of samples defined by the BMQI score.

BMQI score		<0.5	0.5 - <1.4	1.4 - <2.5	2.5 - <3.5	≥3.5	*p*-value
**BM sample pull**	first, n (%)	0 (0.0%)	9 (4.1%)	41 (18.7%)	92 (42.0%)	77 (35.2%)	
	second, n (%)	11 (8.3%)	41 (31.1%)	59 (44.7%)	19 (14.4%)	2 (1.5%)	
**WBC**	(n)	(10)	(46)	(96)	(108)	(76)	<0.001
	WBC, 10^6^/mL	9.9	14.8	22.0	36.8	51.3	
**MGUS**	(n)	(7)	(27)	(42)	(34)	(18)	<0.001
	plasma cells, %	0.084	0.14	0.32	0.73	0.92	
**SMM**	(n)	(3)	(19)	(47)	(64)	(53)	<0.001
	plasma cells, %	0.077	0.68	2.2	2.7	2.7	
**MM**	(n)	(1)	(4)	(11)	(13)	(8)	0.15
	% plasma cells	2.2	2.6	3.7	8.0	5.1	

*BMQI bone marrow quality index, WBC white blood cell, MGUS* monoclonal gammopathy of undetermined significance, *SMM* smoldering multiple myeloma, *MM* multiple myeloma.

**Fig. 5:**
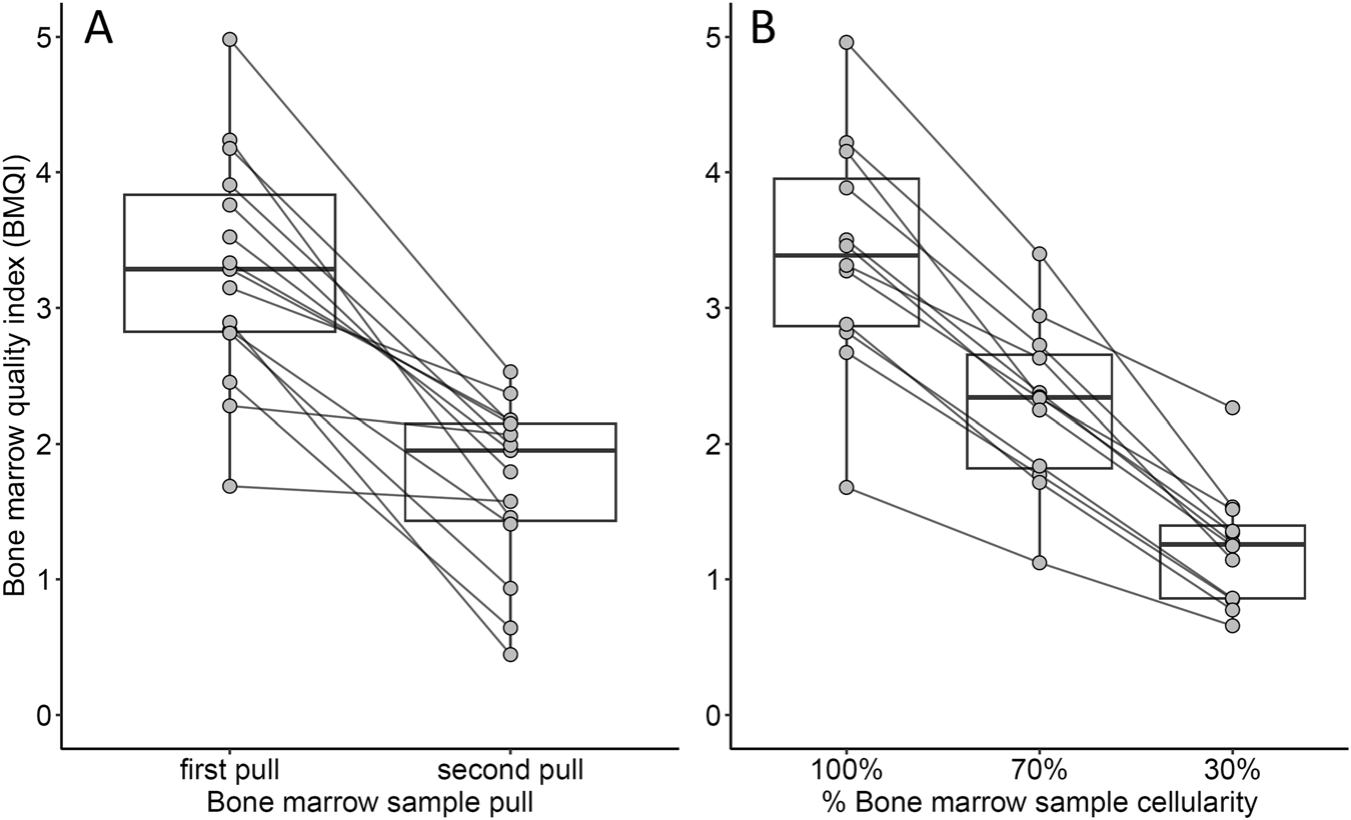
Distribution of the bone marrow quality index (BMQI) in paired first and second pull bone marrow (BM) samples and the effect of dilution of BM by peripheral blood (PB) on BMQI. **A** Distribution of BMQI in paired first pull and second pull BM samples collected during the same aspiration procedure (*n* = 15; *p* < 0.001). **B** Distribution of the BMQI in groups of undiluted (100%) BM aspirated samples and paired samples diluted with pre-defined numbers of blood cells, containing 70% and 30% cellularity of the original BM aspirated sample (*n* = 12; *p* < 0.001 for 100% vs. 30%, *p* < 0.05 for 100% vs 70% and 70% vs. 30%).

To further validate the BMQI as a predictor of hemodilution we compared the BMQI for first pull samples to matched donor second pull samples collected during the same BM aspiration procedure. The first pull BM samples had a higher median BMQI compared to their paired second pull BM samples (3.3 vs. 2.0, respectively; *n* = 15; *p* < 0.001) for all donors (Fig. 5A). Subsequently, we evaluated the effect of an increased blood contamination of first pull BM samples on the BMQI by diluting BM samples with blood based at predefined cell ratios. We observed a gradual decrease in median BMQI values from undiluted samples to 70% BM/30% blood samples, and to 30% BM/70% blood samples (3.4, 2.3, and 1.3, respectively; *n* = 12; *p* < 0.001) (Fig. 5B). The regression analysis revealed a significant relationship between relative changes in the BMQI and the percentage of total plasma cells in diluted samples (estimate = 1.31; *n* = 24; *p* < 0.001). This was further supported by the linear relationship between the BMQI and the proportion of BM sample-derived cellularity and plasma cells in virtual dilutions (Supplementary Fig. 2).

## Discussion

In this prospective study of 351 diagnostic samples from 219 participants in the iStopMM study, we evaluated BM-associated cell populations as indicators of hemodilution. Based on our findings, we developed a novel BMQI, intrinsic to NGF analysis of plasma cells, for objective assessment of hemodilution in BM aspirated samples and their adequacy for analytical use.

In our initial comparison of BM sample pulls, we observed that second pull samples display markedly decreased proportions of plasma cells and other BM-associated cell populations compared to first pull samples. This observation aligns with previous findings highlighting the pronounced increase in hemodilution with sequential BM aspirations and emphasizes the importance of using first pull BM samples for both diagnostic and MRD evaluations [13, 23]. In cases where first pull BM samples are prioritized for other assays than NGF, obtaining a technical first pull sample by needle repositioning has been suggested [14].

Myeloid precursors and nucleated red cells outperformed other cell populations in predicting BM sample pull, suggesting their superior ability to reflect hemodilution in BM aspirates. Interestingly, while the WBC count exhibited an intermediate predictive value of sample pull, it could serve as an easily obtained supplementary measure of sample quality in assays, like NGS, which don’t provide information on BM cellularity. A cutoff for mast cells was initially proposed as indication for BM hemodilution when using NGF [18]. More recently, a paper by the Spanish MM group (GEM) reported treatment-specific and normal reference values for B cell precursors, mast cells, and nucleated red cells for indication of BM hemodilution, but they did not provide guidance about the specific markers and/or criteria of hemodilution [20]. Interestingly, of all markers evaluated in our study, mast cells ranked second to last in predicting BM sample pull, with by far the highest relative variability. Our findings indicate that mast cells are a comparatively poor marker of BM sample quality, contradicting the prevailing notion of mast cells as the most relevant hemodilution marker in NGF analysis [19]. Several studies have reported on evaluation of BM hemodilution in MRD assessment following treatment [12, 19, 24–26]. However, these studies largely rely on arbitrary cutoffs of either mast cells alone or the combination of mast cells and other BM-associated cell populations. Others have reported on flow cytometry-based methods for assessing hemodilution, such as by identifying stages in granulocyte maturation and leveraging their differential distribution between BM and peripheral blood [27–31]. Although these approaches can offer valid assessments, they are not ideal for quality control of BM samples intended for NGF analysis in plasma cell disease, as they lack intrinsic compatibility to the MM specific panel and would require a separate analysis. Together, these findings suggest that myeloid precursors and nucleated red cells are the most reliable indicators of hemodilution occurring in second pull BM samples when using NGF.

Based on the observations above, we formulated the BMQI as a predictive model to assess the degree of hemodilution in BM aspirated samples. The index utilizes the linear combination of myeloid precursors and nucleated red cells, where their associated coefficients represent the weighted contribution in distinguishing the variance between first and second pull BM samples. The performance of the BMQI was subsequently evaluated using a series of prospectively collected samples. Our findings revealed a strong association between the BMQI and BM pull in paired samples and the level of experimental and virtual hemodilution. This multifaceted evaluation underscores the ability of the BMQI to accurately represent the degree of hemodilution in BM aspirated samples. Importantly, given the potential for disease progression to influence the normal cellular composition of the BM, the predictive performance and distribution of the BMQI was consistent across disease stages. Additionally, our findings indicate that the BMQI operates independently of age. These observations reinforce the potential for the usage of the BMQI across different monoclonal gammopathies and age groups. We devised a leveled BMQI scoring system to simplify the interpretation of the BMQI. The interpretive value of this system is underscored by the observed differences in the proportion of plasma cells across the defined groups. We recommend using the BMQI and its scoring system to assess and report BM sample quality. Specifically, a sample with BMQI score of ≥2.5 should be considered of adequate quality, with lower scores indicating increased hemodilution, as follows: <2.5: hemodiluted; <1.4: considerably hemodiluted; and <0.5: severely hemodiluted. For highly hemodiluted samples, resampling may be informed by the BMQI scoring system.

Strengths of this study include the large number of prospectively evaluated samples that were collected by a small team of specialized personnel. Furthermore, the application of standardized and well-established protocols for sample analysis contributes to enhanced reproducibility. The screened cohort reflects the cellular composition in BM across the spectrum of plasma cell neoplasms from MGUS to SMM and MM. Additionally, the thorough follow-up of participants within the iStopMM trial will provide material for future studies with regards to outcomes. The BMQI was validated in both serial aspirate pulls and experimentally diluted samples and provides a novel approach for evaluating quality of BM samples.

The universal limiting factor for evaluating hemodilution in BM aspirated samples is the lack of pure BM controls or well-established hemodilution markers. It is therefore inherently challenging to determine the true distribution of BM-associated cell populations and set relevant reference values to assess hemodilution. Thus, we used the comparison between first and second pull samples as reference for optimal and suboptimal samples. The BMQI was not evaluated with regards to information on outcomes, at the same time, this study did not include individuals who have received MM therapy. Although the BMQI may offer insights into the quality of BM samples after MM treatment, therapy may affect the distribution of the cell populations upon which it is based. Further validation is needed for samples from treated patients. The BMQI is derived based on normal cellularity, which requires the identification of tumor plasma cells to counteract the influence of varied tumor load. Importantly, studies have indicated a robust ability of the MM-MRD for tumor plasma cell detection across different monoclonal gammopathies [32–34].

In summary, we introduce the BMQI as a novel intra-sample quality control for NGF analysis of BM samples in plasma cell diseases. This index combines myeloid precursors and nucleated red cells, which were found to be highly indicative of BM hemodilution. We advocate for the implementation of the BMQI for monitoring of the adequacy of BM samples used in NGF analysis for plasma cell disorders. The BMQI is intrinsic to the EuroFlow MM-MRD assay and can therefore be implemented in flow cytometry analytical software programs for use in all laboratories employing the technique without any change in protocols. It objectively informs about the quality of NGF results, and as such, has the potential to improve the diagnostic and prognostic utility of NGF in plasma cell disorders.

### Supplementary information

Supplemental material

## Data Availability

The datasets generated during and/or analysed during the current study are available from the corresponding author on reasonable request.
