# Supplementary material for: Determining hemodilution in diagnostic bone marrow aspirated samples in plasma cell disorders by next-generation flow cytometry: Proposal for a bone marrow quality index
Source: Blood Cancer J. 2023 Dec 1;13(1):177. doi: 10.1038/s41408-023-00951-2 (PMC10692231; doi:10.1038/s41408-023-00951-2)
Supplement: Supplementary file 1 — Supplemental material [file 41408_2023_951_MOESM1_ESM.docx]

**Supplemental Information**

**Supplemental figures**

**A.**
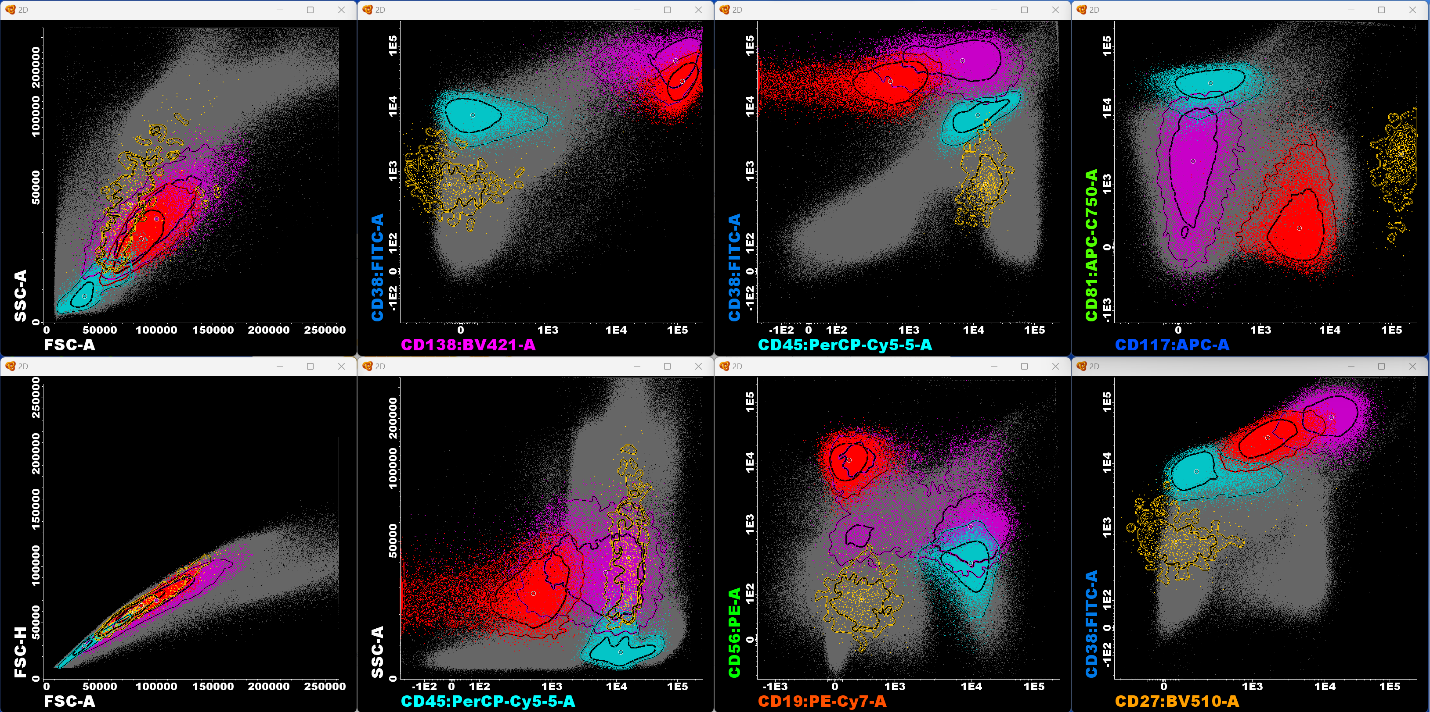


**B.**


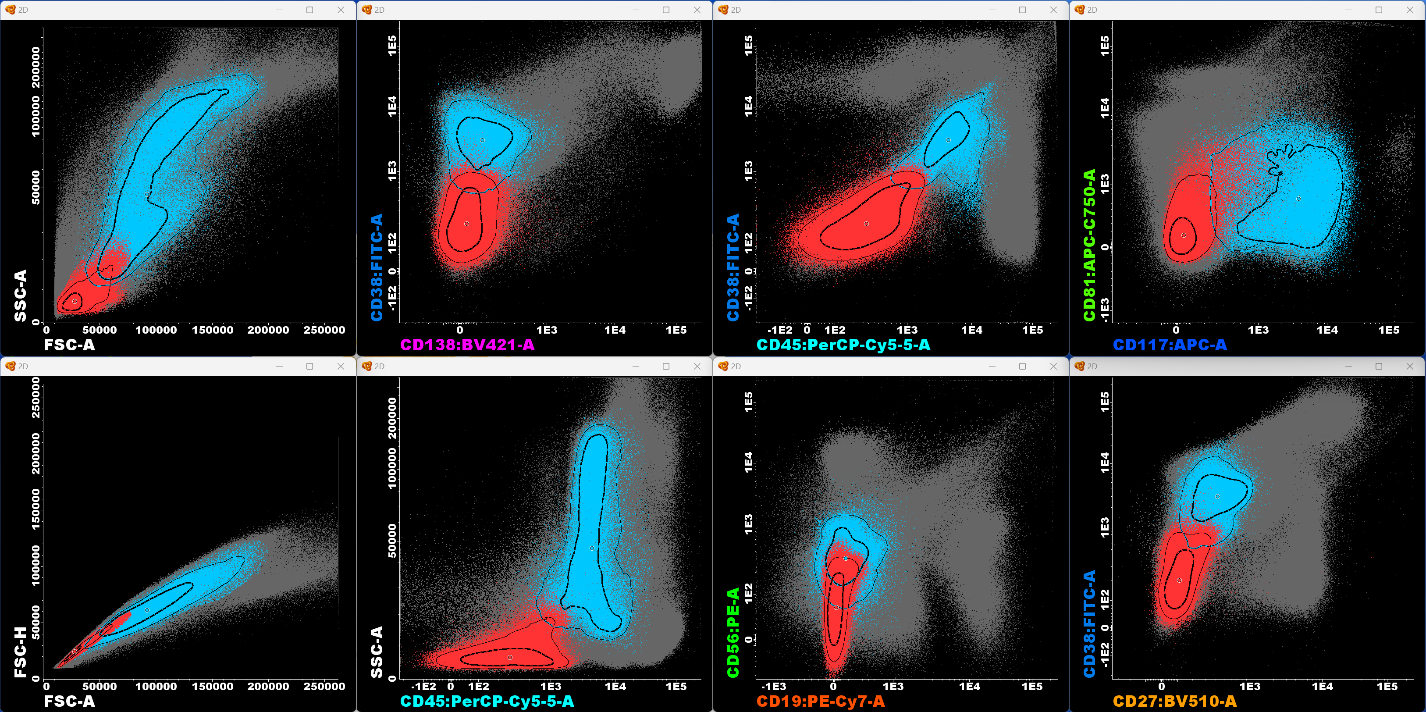


**Supplemental Figure 1: Identification of bone marrow (BM)-associated cell populations in BM aspirated samples analyzed by the EuroFlow multiple myeloma (MM) – minimal residual disease (MRD) panel.** The figure shows reference images of **(A)**: plasma cells (identified by their distinct CD38, CD138, and CD45 expression and light-scatter features), separated into normal (in purple) and abnormal (in red) counterparts; B cell precursors (CD19^+^, CD45^lo^, CD38^hi^, CD81^hi^, and CD27^-^; in turquoise); and mast cells (CD117^high^ and CD45^lo^; in yellow) and **(B):** myeloid precursors (CD117^+^, CD38^+^, CD45^lo^, and SSC^int/hi^ ; in blue) and nucleated red cells (in red; SSC^lo^ and negative expression of all markers) as identified by the AG&I tool coupled with the EuroFlow MM-MRD database in the Infinicyt software. The depicted populations are shown after assigning “check-populations” and removal of debris/doublets. All acquired events, except for the depicted populations, are shown as gray background for each figure. Standard deviation (SD) is shown for each population as 1 SD (inner line) and 2 SD (outer line). Information on the AG&I tool and the EuroFlow MM-MRD database and analysis of fcs files using these features is available on the website of the developer of the Infinicyt software (www.cytognos.com/infinicyt).

**A.**


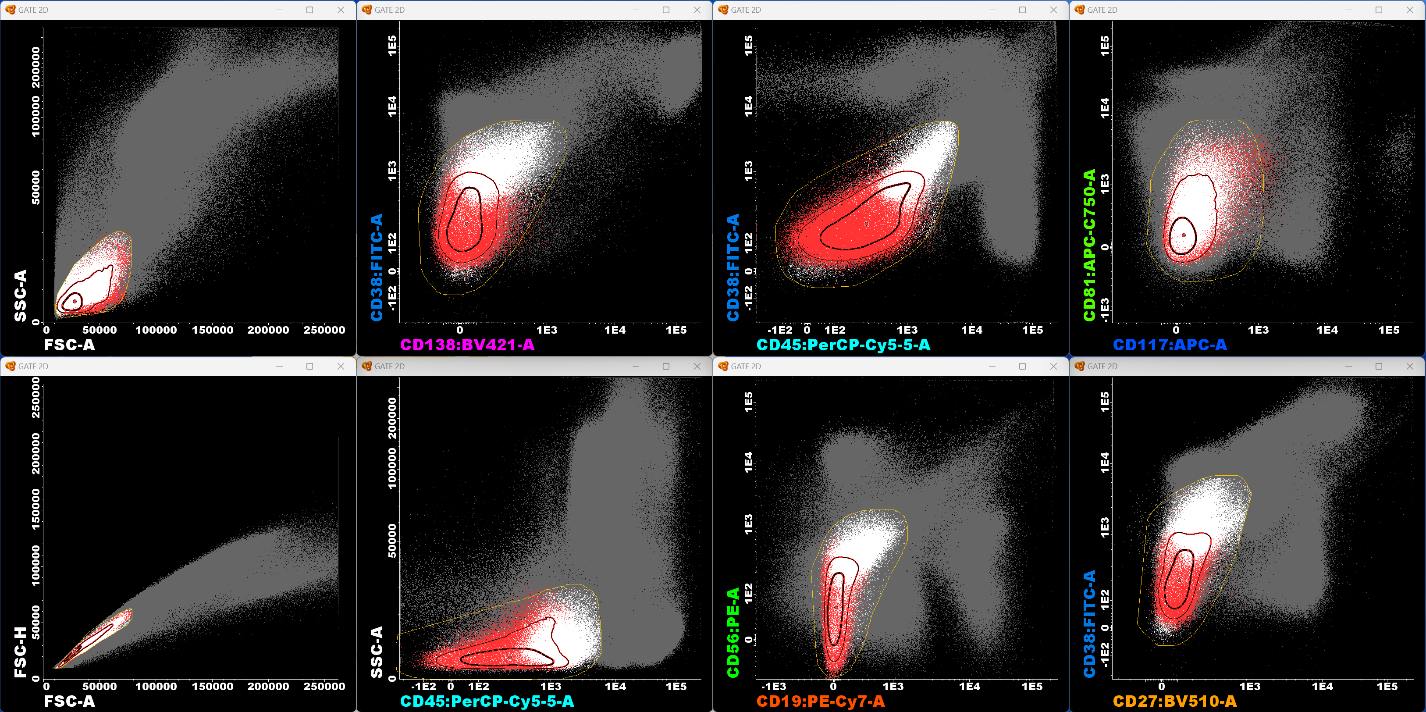


**B.**


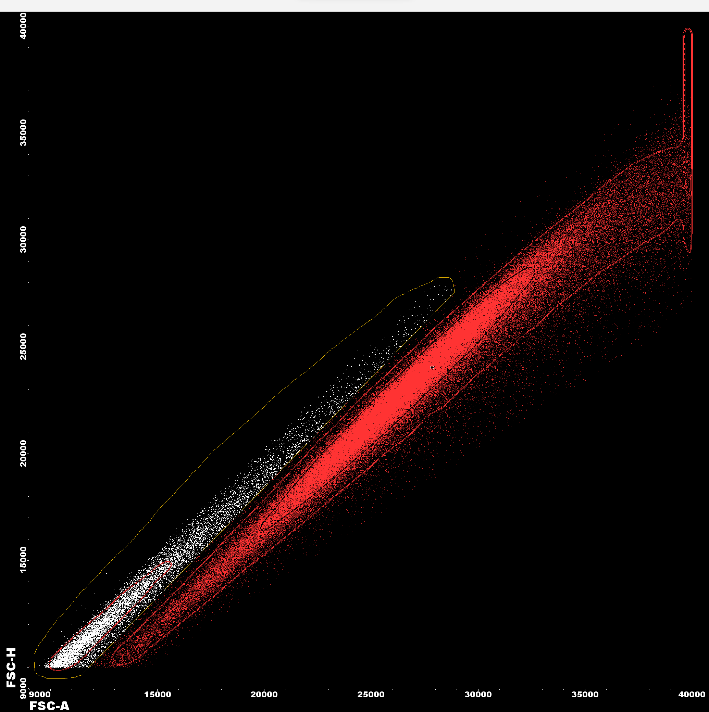

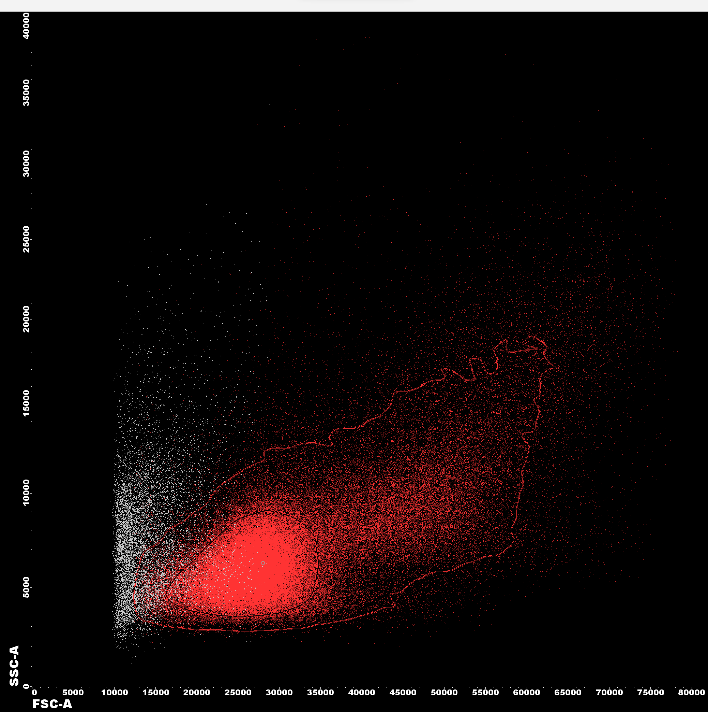


**Supplemental Figure 2. Manual review of the nucleated red cell population after identification by AG&I: (A)** **Gating of residual nucleated red cell events.** When reviewing results of the AG&I feature it is important to be aware of events that may have been mislabeled. The figure show gating of residual nucleated cells using the nucleated red cell population identified by the AG&I tool as reference. **(B) Excluding residual debris from nucleated red cells.** When identifying nucleated red cells it is important to consider that the population is defined by negative expression and low SSC and can therefore be difficult to discriminate from debris. In addition to debris exclusion on FSC-A vs SSC-A, gating on FSC-A vs FSC-H can aid in identifying residual debris, as shown in the figure on the left (the figure on the right shows gated events on a FSC-A vs SSC-A for reference). This gate can be emphasized using a zoom function, such as available in the Infinicyt software.


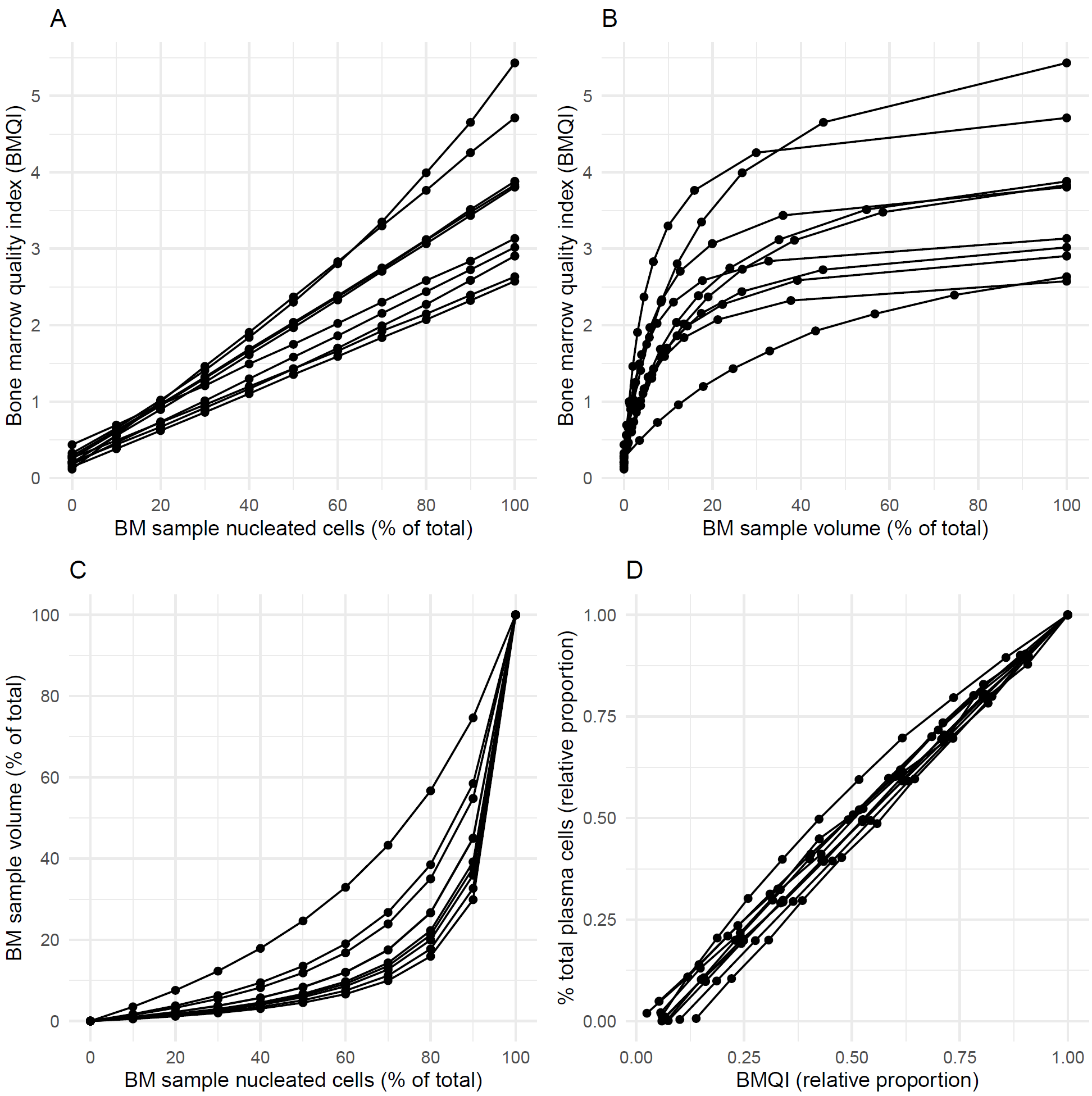


**Supplemental figure 2. Virtual dilution analysis of BM and PB samples: Assessing the cellular and volumetric impacts of varying degrees of BM hemodilution on the bone marrow quality index (BMQI). (A)** Impact of increasing proportion of nucleated cells derived from the bone marrow sample (as percentage of total) on the BMQI across the dilution series. Median BMQI of 0.018, 0.16, 0.32, 0.48, 0.64, 0.80, 0.96, 1.13, 1.27, 1.45, and 1.62, from 0% to 100% BM sample nucleated cells, respectively (r=0.92; p<0.001) **(B)** Impact of increasing proportion of the bone marrow sample volume (as percentage of the total) on the BMQI across the dilution series. **(C)** Relationship between the cellular and volumetric proportions of the original bone marrow sample across the dilution series. Median % BM sample volume of 0.0, 0.79, 1.76, 2.98, 4.55, 6.68, 9.96, 14.31, 22.25, 39.17, and 100, from 0% to 100% BM sample nucleated cells, respectively **(D)** Correlation between the proportion of the BMQI and the percentage of total plasma cells in the dilution series, relative to the undiluted bone marrow sample (r=0.99; p<0.001). Each graph is presented as a scatterplot with data points for each dilution sequentially connected, representing individual donors.

**Supplemental methods**

**Virtual dilution of bone marrow samples**

Virtual dilutions were performed using prospectively collected paired bone marrow (BM) and peripheral blood (PB) samples from 10 individuals. The dilutions were simulated based on the pre-determined number of nucleated cellular events in the paired samples, obtained from participants of the Iceland Screens, Treats, or Prevents Multiple Myeloma study (iStopMM), diagnosed with monoclonal gammopathy of undetermined significance (MGUS; n=3), smoldering multiple myeloma (SMM; n=4), and multiple myeloma (MM; n=3). Initially, BM and PB samples were prepared and stained separately using the antibody combination from the EuroFlow – minimal residual disease (MRD) panel according to EuroFlow protocols as described in the paper. Subsequently, dilutions were simulated in the Infinicyt software by merging a specified number of nucleated cellular events from the paired BM and PB flow cytometry standard (FCS) files. This produced a series of FCS files, each containing a predetermined percentage of nucleated cellular events from the original BM FCS file, ranging from 0% to 100% in 10% increments (11 files in total). For each donor, the series was merged into a single file, with distinct identification for individual files. The merged files were gated manually in full, ensuring consistent gating across all dilutions in the series for each donor. Population percentages were determined for each file in the dilutions series separately and used to calculate the bone marrow quality index (BMQI). Additionally, nucleated cellular events from BM and PB in each file were used to compute the corresponding sample volume. This was achieved using a dual-platform approach that utilized the pre-analytical white blood cell (WBC) count of the BM and PB samples. Pearson’s correlation was used to assess the relationship between two variables in the dilution series.
